# Supplementary material for: Genomic diversity, host associations, and tissue tropism of Hydrangea ringspot virus: A global and regional perspective
Source: PLoS One. 2026 Oct 8;21(10):e0348969. doi: 10.1371/journal.pone.0348969 (PMC13649025; doi:10.1371/journal.pone.0348969)
Supplement: Peer review history

# Peer review: Decision Letter 0

**DOI:** 10.1371/journal.pone.0348969.r001

**Authors:** Mário Ferreira Conceição Santos

27 Jul 2026

Thank you for submitting your manuscript to PLOS ONE. After careful consideration, we feel that it has merit but does not fully meet PLOS ONE’s publication criteria as it currently stands. Therefore, we invite you to submit a revised version of the manuscript that addresses the points raised during the review process.

Please submit your revised manuscript by Sep 10 2026 11:59PM. If you will need more time than this to complete your revisions, please reply to this message or contact the journal office at plosone@plos.org. When you're ready to submit your revision, log on to https://www.editorialmanager.com/pone/ and select the 'Submissions Needing Revision' folder to locate your manuscript file.

- A letter that responds to each point raised by the academic editor and reviewer(s). You should upload this letter as a separate file labeled 'Response to Reviewers'.
- A marked-up copy of your manuscript that highlights changes made to the original version. You should upload this as a separate file labeled 'Revised Manuscript with Track Changes'.
- An unmarked version of your revised paper without tracked changes. You should upload this as a separate file labeled 'Manuscript'.

If you would like to make changes to your financial disclosure, please include your updated statement in your cover letter. Guidelines for resubmitting your figure files are available below the reviewer comments at the end of this letter.

If applicable, we recommend that you deposit your laboratory protocols in protocols.io to enhance the reproducibility of your results. Protocols.io assigns your protocol its own identifier (DOI) so that it can be cited independently in the future. For instructions see: https://journals.plos.org/plosone/s/submission-guidelines#loc-laboratory-protocols. Additionally, PLOS ONE offers an option for publishing peer-reviewed Lab Protocol articles, which describe protocols hosted on protocols.io. Read more information on sharing protocols at https://plos.org/protocols?utm_medium=editorial-email&utm_source=authorletters&utm_campaign=protocols.

As the corresponding author, your ORCID iD is verified in the submission system and will appear in the published article. PLOS supports the use of ORCID, and we encourage all coauthors to register for an ORCID iD and use it as well. Please encourage your coauthors to verify their ORCID iD within the submission system before final acceptance, as unverified ORCID iDs will not appear in the published article. *Only*  the individual author can complete the verification step; PLOS staff *cannot*  verify ORCID iDs on behalf of authors.

We look forward to receiving your revised manuscript.

Kind regards,

Mário Ferreira Conceição Santos

Academic Editor

PLOS One

Journal Requirements:

When submitting your revision, we need you to address these additional requirements.

1. Please ensure that your manuscript meets PLOS ONE's style requirements, including those for file naming. The PLOS ONE style templates can be found at

https://journals.plos.org/plosone/s/file?id=wjVg/PLOSOne_formatting_sample_main_body.pdf and

https://journals.plos.org/plosone/s/file?id=ba62/PLOSOne_formatting_sample_title_authors_affiliations.pdf

2. In your Methods section, please provide additional information regarding the permits you obtained for the work. Please ensure you have included the full name of the authority that approved the field site access and, if no permits were required, a brief statement explaining why

3. Please note that PLOS One has specific guidelines on code sharing for submissions in which author-generated code underpins the findings in the manuscript. In these cases, we expect all author-generated code to be made available without restrictions upon publication of the work. Please review our guidelines at https://journals.plos.org/plosone/s/materials-and-software-sharing#loc-sharing-code and ensure that your code is shared in a way that follows best practice and facilitates reproducibility and reuse.

4. Please remove your figures from within your manuscript file, leaving only the individual TIFF/EPS image files, uploaded separately. These will be automatically included in the reviewers’ PDF.

5. We note that Figure 1 in your submission contain copyrighted images. All PLOS content is published under the Creative Commons Attribution License (CC BY 4.0), which means that the manuscript, images, and Supporting Information files will be freely available online, and any third party is permitted to access, download, copy, distribute, and use these materials in any way, even commercially, with proper attribution. For more information, see our copyright guidelines: http://journals.plos.org/plosone/s/licenses-and-copyright.

We require you to either (1) present written permission from the copyright holder to publish these figures specifically under the CC BY 4.0 license, or (2) remove the figures from your submission:

1. You may seek permission from the original copyright holder of Figure 1 to publish the content specifically under the CC BY 4.0 license.

We recommend that you contact the original copyright holder with the Content Permission Form (http://journals.plos.org/plosone/s/file?id=7c09/content-permission-form.pdf) and the following text:

“I request permission for the open-access journal PLOS ONE to publish XXX under the Creative Commons Attribution License (CCAL) CC BY 4.0 (http://creativecommons.org/licenses/by/4.0/). Please be aware that this license allows unrestricted use and distribution, even commercially, by third parties. Please reply and provide explicit written permission to publish XXX under a CC BY license and complete the attached form.”

Please upload the completed Content Permission Form or other proof of granted permissions as an "Other" file with your submission.

In the figure caption of the copyrighted figure, please include the following text: “Reprinted from [ref] under a CC BY license, with permission from [name of publisher], original copyright [original copyright year].”

2. If you are unable to obtain permission from the original copyright holder to publish these figures under the CC BY 4.0 license or if the copyright holder’s requirements are incompatible with the CC BY 4.0 license, please either i) remove the figure or ii) supply a replacement figure that complies with the CC BY 4.0 license. Please check copyright information on all replacement figures and update the figure caption with source information. If applicable, please specify in the figure caption text when a figure is similar but not identical to the original image and is therefore for illustrative purposes only.

6.When completing the data availability statement of the submission form, you indicated that you will make your data available on acceptance. We strongly recommend all authors decide on a data sharing plan before acceptance, as the process can be lengthy and hold up publication timelines. Please note that, though access restrictions are acceptable now, your entire data will need to be made freely accessible if your manuscript is accepted for publication. This policy applies to all data except where public deposition would breach compliance with the protocol approved by your research ethics board. If you are unable to adhere to our open data policy, please kindly revise your statement to explain your reasoning and we will seek the editor's input on an exemption. Please be assured that, once you have provided your new statement, the assessment of your exemption will not hold up the peer review process.

7. If the reviewer comments include a recommendation to cite specific previously published works, please review and evaluate these publications to determine whether they are relevant and should be cited. There is no requirement to cite these works unless the editor has indicated otherwise.

[Note: HTML markup is below. Please do not edit.]

Reviewers' comments:

Reviewer's Responses to Questions

**Comments to the Author**

1. Is the manuscript technically sound, and do the data support the conclusions?

Reviewer #1: Yes

**********

2. Has the statistical analysis been performed appropriately and rigorously?

Reviewer #1: Yes

**********

3. Have the authors made all data underlying the findings in their manuscript fully available?

The PLOS Data policy

Reviewer #1: Yes

**********

4. Is the manuscript presented in an intelligible fashion and written in standard English?

Reviewer #1: Yes

**********

Reviewer #1: This manuscript provides a comprehensive global analysis of Hydrangea ringspot virus (HdRSV, Potexvirus hydrangea) by coupling field sampling in Colombia with an extensive in silico screening of 210 public Hydrangea transcriptomes. The authors successfully assembled 23 novel viral sequences, including the first genomic report of HdRSV from South America (isolate UdeA), and identified two distinct global lineages (HdRSV-L1 and HdRSV-L2). Additionally, the study maps tissue tropism, showing high viral accumulation in roots, and identifies a fixed non-synonymous mutation (Thr526Ile) that distinguishes the two lineages.

The paper addresses a significant gap in ornamental plant virology and effectively leverages public data. However, the manuscript contains an excessive number of figures, and several methodological assumptions regarding host comparisons and tissue tropism require revision.

Major Comments

The current draft contains too many figures, many of which share similar results and disrupt the narrative flow. Please consolidate them into multi-panel figures organized by biological themes:

Isolate Characterization: Combine Figure 1 (symptoms) and Figure 2 (coverage/GC content) into a single multi-panel figure.

Phylogenetics: Merge individual gene trees and the whole-genome tree into one multi-panel figure, or move individual gene trees to the Supplementary Materials.

Viral Quantification: Group the separate tissue tropism and host/species comparison plots into a single figure with panels A and B.

Mutation Analysis: Combine the sequence variant mapping with Figure 14 (the Thr526Ile structural mutation visualization).

The conclusion that viral load is significantly higher in H. serrata than in H. macrophylla is compromised because all H. serrata data came from a single cultivar (Oamacha). This finding must be qualified throughout the text and abstract as a cultivar-specific difference rather than a broad species-wide trait.

The Colombian isolate UdeA covers 70% of the genome. Please ensure that terms like reconstructing the complete viral genome are qualified accurately. Additionally, the temporary Accession number pending placeholders must be replaced with final GenBank/SRA identifiers before formal acceptance.

The high relative accumulation (TPM) of a non-soil-borne virus in roots is interesting. However, please discuss whether this could be partially driven by a lower baseline of host housekeeping transcripts in root tissues relative to leaves, which might artificially inflate relative TPM values.

Figure 2 highlights a drop in coverage overlapping with a high GC content peak. Please clarify if this systematic bias was the primary factor preventing full-genome resolution across the other public SRA datasets.

Minor Comments

In the introduction, correct the phrase renaming the specie... to renaming the species....

Standardize the in-text citations by replacing the Spanish coordinate conjunction y with the English and (e.g., change Parrella y Vovlas to Parrella and Vovlas).

Ensure consistency when referencing supplementary files, aligning text mentions (e.g., S1 Fig) with the specific formatting guidelines of the journal.

The text caption for Figure 1 mentions yellow and red arrows, but these are missing from the figure itself. Please ensure all described annotations are visible in the final graphics.

In Figure 14, update the label Thr > Ile 526 to include the corresponding nucleotide change or absolute genomic coordinate for clearer reference.

**********

what does this mean?). If published, this will include your full peer review and any attached files.

If you choose “no”, your identity will remain anonymous but your review may still be made public.

**Do you want your identity to be public for this peer review?**  For information about this choice, including consent withdrawal, please see our Privacy Policy

Reviewer #1: **Yes:**  Won Kyong Cho

**********

[NOTE: If reviewer comments were submitted as an attachment file, they will be attached to this email and accessible via the submission site. Please log into your account, locate the manuscript record, and check for the action link "View Attachments". If this link does not appear, there are no attachment files.]

To ensure your figures meet our technical requirements, please review our figure guidelines: https://journals.plos.org/plosone/s/figures

You may also use PLOS’s free figure tool, NAAS, to help you prepare publication quality figures: https://journals.plos.org/plosone/s/figures#loc-tools-for-figure-preparation.

NAAS will assess whether your figures meet our technical requirements by comparing each figure against our figure specifications.

# Author comment: Author response to Decision Letter 1

**DOI:** 10.1371/journal.pone.0348969.r002

8 Sep 2026

PONE-D-26-20250

Genomic Diversity, Host Associations, and Tissue Tropism of Hydrangea Ringspot Virus: A Global and Regional Perspective

PLOS One

1. Please ensure that your manuscript meets PLOS ONE's style requirements, including those for file naming.

R: The manuscript was revised to comply with PLOS ONE requirements.

2. In your Methods section, please provide additional information regarding the permits you obtained for the work. Please ensure you have included the full name of the authority that approved the field site access and, if no permits were required, a brief statement explaining why

R: We have added the following statement to the Methods section (2.1): " Access to the commercial production site was authorized directly by the farm owner, who requested to remain anonymous. As sampling was conducted on privately cultivated ornamental plants within a commercial greenhouse operation, rather than in a protected or wild area, no additional environmental or governmental permits were required.

3. Please note that PLOS One has specific guidelines on code sharing for submissions in which author-generated code underpins the findings in the manuscript. In these cases, we expect all author-generated code to be made available without restrictions upon publication of the work.

R: No specific code was developed for this work. All bioinformatics analyses were performed using publicly available software and established bioinformatics tools, and the corresponding references and citations are provided in the manuscript. Therefore, no author-generated code is associated with the analyses presented in this study.

4. Please remove your figures from within your manuscript file, leaving only the individual TIFF/EPS image files, uploaded separately. These will be automatically included in the reviewers’ PDF.

R: All figures have been removed from the manuscript file and will be uploaded separately as individual TIFF files, following the new figure numbering and panel consolidation described above (Fig 1–Fig 6, plus S1–S3 Fig). Only the figure captions remain within the manuscript text, placed together at the end of the file as required.

5. We note that Figure 1 in your submission contain copyrighted images. All PLOS content is published under the Creative Commons Attribution License (CC BY 4.0), which means that the manuscript, images, and Supporting Information files will be freely available online, and any third party is permitted to access, download, copy, distribute, and use these materials in any way, even commercially, with proper attribution. For more information, see our copyright guidelines: http://journals.plos.org/plosone/s/licenses-and-copyright.

R: We confirm that the photograph in Fig 1 (panel A) is original, unpublished material taken by the authors at our own facilities and is not copyrighted third-party content. No permission is required, and we retain full rights to publish this image under the CC BY 4.0 license.

6. When completing the data availability statement of the submission form, you indicated that you will make your data available on acceptance. We strongly recommend all authors decide on a data sharing plan before acceptance, as the process can be lengthy and hold up publication timelines. Please note that, though access restrictions are acceptable now, your entire data will need to be made freely accessible if your manuscript is accepted for publication. This policy applies to all data except where public deposition would breach compliance with the protocol approved by your research ethics board. If you are unable to adhere to our open data policy, please kindly revise your statement to explain your reasoning and we will seek the editor's input on an exemption. Please be assured that, once you have provided your new statement, the assessment of your exemption will not hold up the peer review process.

R: We have updated our Data Availability Statement accordingly. All sequence data generated in this study have now been deposited in the European Nucleotide Archive (ENA) under project accession PRJEB101813 (ERP183217) and are publicly available, including the Colombian HdRSV isolate UdeA (accession OZ367897). No exemption is required

Reviewers' comments:

Review Comments to the Author

• Reviewer #1: This manuscript provides a comprehensive global analysis of Hydrangea ringspot virus (HdRSV, Potexvirus hydrangea) by coupling field sampling in Colombia with an extensive in silico screening of 210 public Hydrangea transcriptomes. The authors successfully assembled 23 novel viral sequences, including the first genomic report of HdRSV from South America (isolate UdeA), and identified two distinct global lineages (HdRSV-L1 and HdRSV-L2). Additionally, the study maps tissue tropism, showing high viral accumulation in roots, and identifies a fixed non-synonymous mutation (Thr526Ile) that distinguishes the two lineages. The paper addresses a significant gap in ornamental plant virology and effectively leverages public data. However, the manuscript contains an excessive number of figures, and several methodological assumptions regarding host comparisons and tissue tropism require revision.

R: We thank the reviewer for the positive assessment of our study and for the constructive feedback. We have substantially reduced the number of figures by consolidating thematically related panels (from 14 to 6 main figures, with additional detail moved to the Supporting Information), and we have revised the text to appropriately qualify the host comparison and tissue tropism findings, as detailed in our point-by-point responses below.

Major Comments

The current draft contains too many figures, many of which share similar results and disrupt the narrative flow. Please consolidate them into multi-panel figures organized by biological themes:

• Isolate Characterization: Combine Figure 1 (symptoms) and Figure 2 (coverage/GC content) into a single multi-panel figure.

R: Figures 1 and 2 have been merged into a single multi-panel figure (new Fig 1), with panel A showing the morphological alterations of the Colombian plant, panel B showing GC content across the HdRSV reference genome, and panel C showing sequencing coverage across the same genome. The caption now reads: "Morphological and genomic characterization of the Colombian HdRSV isolate. (A) Morphological alterations observed in a Colombian Hydrangea macrophylla plant. Leaves display irregular necrotic spotting together with reddish-purple discoloration along the margins and interveinal areas. The apical inflorescences remain small, compact, and undeveloped. (B) GC content (%) calculated in sliding windows of 200 bp with 20 bp step size along the HdRSV reference genome (NC_006943), showing pronounced GC-rich regions. (C) Percentage of HdRSV-positive samples with sequencing coverage at each viral genome position, based on read mapping to the reference genome."

• Phylogenetics: Merge individual gene trees and the whole-genome tree into one multi-panel figure, or move individual gene trees to the Supplementary Materials.

R: We thank the reviewer for this suggestion. Our manuscript does not include separate single-gene trees — only a main-text tree based on complete coding sequences (now Fig 2) and a supplementary tree based on the replicase CDS (S1 Fig), which already follows the structure the reviewer proposes.

• Viral Quantification: Group the separate tissue tropism and host/species comparison plots into a single figure with panels A and B.

R: We have consolidated the requested plots into a single two-panel figure: panel A shows viral tissue tropism (formerly Fig 9) and panel B shows the host species/lineage comparison (formerly Fig 11). In addition, and in the same spirit of reducing figure redundancy, we proactively merged the prevalence plots (formerly Figs 5, 6, 7, and 8) into a single multi-panel "Prevalence" figure.

• Mutation Analysis: Combine the sequence variant mapping with Figure 14 (the Thr526Ile structural mutation visualization).

R: We have substantially reduced the number of figures, as suggested by the reviewer. As a result, the original Figure 14 has been renumbered as Figure 6 and is now the final figure in the manuscript.

• The conclusion that viral load is significantly higher in H. serrata than in H. macrophylla is compromised because all H. serrata data came from a single cultivar (Oamacha). This finding must be qualified throughout the text and abstract as a cultivar-specific difference rather than a broad species-wide trait.

R: We have qualified this finding in the Results section. The sentence now reads: "A direct comparison revealed that the overall median viral load was significantly higher in H. serrata than in H. macrophylla (p = 6.614e-15, Wilcoxon rank sum test with continuity correction); however, as all H. serrata data derived from this single cultivar, this difference should be interpreted as cultivar-specific rather than representative of the species as a whole." The Discussion already included this caveat.

• The Colombian isolate UdeA covers 70% of the genome. Please ensure that terms like reconstructing the complete viral genome are qualified accurately. Additionally, the temporary Accession number pending placeholders must be replaced with final GenBank/SRA identifiers before formal acceptance. R: We confirm that the manuscript consistently refers to the UdeA isolate as a partial genome (70% coverage) and does not describe it as complete elsewhere in the text. The accession placeholder has been replaced: the sequence has been deposited in the European Nucleotide Archive (ENA) under the name HdRSV isolate UdeA (accession OZ367897).

• The high relative accumulation (TPM) of a non-soil-borne virus in roots is interesting. However, please discuss whether this could be partially driven by a lower baseline of host housekeeping transcripts in root tissues relative to leaves, which might artificially inflate relative TPM values.

R: We have added a statement acknowledging this as a limitation of the TPM metric. The Discussion now reads: "The elevated relative TPM values observed in roots should be interpreted with caution, as TPM is a compositional metric sensitive to the overall transcriptional complexity of the sample. Root tissue is generally characterized by a lower diversity and abundance of housekeeping transcripts compared to metabolically more active tissues such as leaves, which could partially inflate the relative proportion attributed to viral transcripts independent of true differences in viral accumulation”.

• Figure 2 highlights a drop in coverage overlapping with a high GC content peak. Please clarify if this systematic bias was the primary factor preventing full-genome resolution across the other public SRA datasets.

R: We have added a statement clarifying this point. The manuscript now reads: "This coverage gap coincided with a locally elevated GC content region of the HdRSV genome (Fig 2), suggesting that GC-associated sequencing bias — rather than low viral titer or insufficient depth — was likely the primary factor limiting full-genome resolution both in our sample and across the public SRA datasets analyzed

Minor Comments

• In the introduction, correct the phrase renaming the specie... to renaming the species.

R: We thank the reviewer for catching this typo. It has been corrected in the Introduction accordingly.

• Standardize the in-text citations by replacing the Spanish coordinate conjunction y with the English and (e.g., change Parrella y Vovlas to Parrella and Vovlas).

R: Corrected throughout the manuscript.

• Ensure consistency when referencing supplementary files, aligning text mentions (e.g., S1 Fig) with the specific formatting guidelines of the journal.

R: Thank you for this observation. We have carefully reviewed the manuscript and ensured that all in-text references to supplementary files (e.g., S1 Fig, S2 Fig, S3 Fig, S1 Table) are now consistent with each other and follow the journal's specific formatting guidelines throughout the text.

• The text caption for Figure 1 mentions yellow and red arrows, but these are missing from the figure itself. Please ensure all described annotations are visible in the final graphics.

R: The caption was revised to remove the arrow references and to describe only the features visible in the image. The new caption reads: "Morphological alterations observed in a Colombian Hydrangea macrophylla plant. Leaves display irregular necrotic spotting together with reddish-purple discoloration along the margins and interveinal areas. The apical inflorescences remain small, compact, and undeveloped."

• In Figure 14, update the label Thr > Ile 526 to include the corresponding nucleotide change or absolute genomic coordinate for clearer reference.

R: We have updated the mutation label in Fig 14 -Now Fig 6 to include the corresponding nucleotide change, which now reads "526, Thr > Ile (C1578T)", consistent with the nucleotide position already reported in the main text and Abstract. This allows the mutation to be unambiguously located at both the nucleotide and amino acid levels.

Attachment Submitted filename: R1_Response to Reviewers_Hydrangea.docx

# Editor report: Decision Letter 1

**DOI:** 10.1371/journal.pone.0348969.r003

**Authors:** Mário Ferreira Conceição Santos

23 Sep 2026

Genomic diversity, host associations, and tissue tropism of Hydrangea ringspot virus: A global and regional perspective

PONE-D-26-20250R1

Dear Dr. Juan F. Alzate,

We’re pleased to inform you that your manuscript has been judged scientifically suitable for publication and will be formally accepted for publication once it meets all outstanding technical requirements.

Within one week, you’ll receive an e-mail detailing the required amendments. When these have been addressed, you’ll receive a formal acceptance letter and your manuscript will be scheduled for publication.

An invoice will be generated when your article is formally accepted. Please note, if your institution has a publishing partnership with PLOS and your article meets the relevant criteria, all or part of your publication costs will be covered. Please make sure your user information is up-to-date by logging into Editorial Manager at Editorial Manager® and clicking the ‘Update My Information' link at the top of the page. For questions related to billing, please contact billing support.

If your institution or institutions have a press office, please notify them about your upcoming paper to help maximize its impact. If they’ll be preparing press materials, please inform our press team as soon as possible -- no later than 48 hours after receiving the formal acceptance. Your manuscript will remain under strict press embargo until 2 pm Eastern Time on the date of publication. For more information, please contact onepress@plos.org.

Kind regards,

Mário Ferreira Conceição Santos

Academic Editor

PLOS One

Additional Editor Comments (optional):

Reviewers' comments:

# Editor report: Acceptance letter

**DOI:** 10.1371/journal.pone.0348969.r004

**Authors:** Mário Ferreira Conceição Santos

PONE-D-26-20250R1

PLOS One

Dear Dr. Alzate,

I'm pleased to inform you that your manuscript has been deemed suitable for publication in PLOS One. Congratulations! Your manuscript is now being handed over to our production team.

At this stage, our production department will prepare your paper for publication. This includes ensuring the following:

* All references, tables, and figures are properly cited

* All relevant supporting information is included in the manuscript submission,

* There are no issues that prevent the paper from being properly typeset

You will receive further instructions from the production team, including instructions on how to review your proof when it is ready. Please keep in mind that we are working through a large volume of accepted articles, so please give us a few days to review your paper and let you know the next and final steps.

Lastly, if your institution or institutions have a press office, please let them know about your upcoming paper now to help maximize its impact. If they'll be preparing press materials, please inform our press team within the next 48 hours. Your manuscript will remain under strict press embargo until 2 pm Eastern Time on the date of publication. For more information, please contact onepress@plos.org.

You will receive an invoice from PLOS for your publication fee after your manuscript has reached the completed accept phase. If you receive an email requesting payment before acceptance or for any other service, this may be a phishing scheme. Learn how to identify phishing emails and protect your accounts at https://explore.plos.org/phishing.

If we can help with anything else, please email us at customercare@plos.org.

Thank you for submitting your work to PLOS One and supporting open access.

Kind regards,

PLOS One Editorial Office Staff

on behalf of

Dr. Mário Ferreira Conceição Santos

Academic Editor

PLOS One
